# Sporotrichin Skin Test for the Diagnosis of Sporotrichosis

**DOI:** 10.3390/jof4020055

**Published:** 2018-05-09

**Authors:** Alexandro Bonifaz, Conchita Toriello, Javier Araiza, Max C. Ramírez-Soto, Andrés Tirado-Sánchez

**Affiliations:** 1 Dermatology Service & Mycology Department, Hospital General de México, “Dr. Eduardo Liceaga”, Balmis 148, Colonia Doctores, Ciudad de México 06726, Mexico; javier.araiza55@gmail.com; 2 Departamento de Microbiología y Parasitología, Facultad de Medicina, Universidad Nacional Autónoma de México, Ciudad de México 04510, Mexico; toriello@unam.mx; 3 Medicine School, Universidad Nacional Mayor de San Marcos, Lima 15081, Peru; maxcrs22@gmail.com; 4 Internal Medicine Department, Hospital General de Zona 29, Instituto Mexicano del Seguro Social, Ciudad de México 07950, Mexico

**Keywords:** sporotrichosis, sporotrichin, skin test, *Sporothrix schenckii*

## Abstract

Sporotrichosis is the most common implantation mycosis caused by several species of the *Sporothrix schenckii* complex. The gold standard for diagnosis is concerned with the isolation of the fungus; although, fresh examinations, staining, and biopsies are also helpful for this purpose. The sporotrichin is an antigenic complex comprised of a peptide-rhamnomannan, which is relevant with respect to pathogenic fungi; it is primarily used for serological and skin testing. We present a study regarding the use of sporotrichin as a diagnostic aid for cutaneous sporotrichosis. Furthermore, 138 cases with suspicion of sporotrichosis were included, 55 of which were proven through cultures. Moreover, out of these 55 cases, 52 (94.5%) tested positive for sporotrichin, while the negative cases corresponded to the disseminated cutaneous forms. We observed a sensitivity of 94.5% and a specificity of 95.2%. We consider that the use of sporotrichin as a skin test helps us as an auxiliary diagnosis before a positive sample culture.

## 1. Introduction

Sporotrichosis is a subacute, subcutaneous mycosis that is related to the *Sporothrix* complex, which is primarily represented by *Sporothrix schenckii* (*sensu stricto*). This infection generally develops after a traumatic inoculation of the fungus. It is the most widely-distributed mycosis, occurring due to implantation [1,2,3] in the world, especially in tropical and subtropical regions [1,4,5]. *Sporothrix schenckii* is a dimorphic fungus (mycelial and yeast forms) that, along with the other species that constitute the complex, generally thrives in temperate and humid climates with an average temperature of 20–25 °C, in conjunction with high humidity. It has been isolated from soil, plants, decaying plant, wood, and decomposing matter [1,2,4,5,6,7].

The gold standard for the diagnosis of sporotrichosis is fungus isolation from cutaneous nodules, pus, exudate, or swabbing’s from open lesions, deposited in Sabouraud dextrose-agar; potato dextrose agar can also be used for culture. After five to seven days of culture medium incubation at 26 °C, the mycelial typical morphology of fine septate hyphae with conidiophores that form sympodially ovate conidia with an arrangement that suggests a flower arrangement is observed after a few days [1,2,3,5]. It is important to mention that parasitic yeast structures known as asteroid bodies are observed in fresh examinations (KOH) or in histopathological stains, and they generally provide precise information within a few minutes. However, in most cases concerning sporotrichosis (lymphatic and fixed), these elements are not observed, or are very limited (yeast), which results in a negative report. Histopathology is not very helpful in this regard, because in most cases of fresh examination, yeasts are not observed; in our experience, they are positive (fresh examination under KOH) only between 5–7%. Meanwhile, in a report provided by Quintella et al. [8], they observed that 35% of the sporotrichosis cases were positive.

The antigen of *S. schenckii* was originally extracted as a crude polysaccharide fragment, and has been used as an intradermal reaction, in addition to being used for a few serological tests [9,10]. Sporotrichin has fallen into disuse as a diagnostic test in several countries where it is not available, since no commercial or standardized antigen is available. However, according to our perspective, we believe that it is a useful tool that provides suggestive diagnostic data in a short period of time (48 h average), before culture allows us to determine a definitive diagnosis.

We aimed to present our experience by employing a sporotrichin skin test from the Universidad Nacional Autónoma de México, which was previously studied in an endemic Mexican community that showed a 53.2% positive reaction [11]; this time, it will be used as an aid for the diagnosis of sporotrichosis.

## 2. Materials and Methods

### 2.1. Baseline Characteristics of the Study Population

Over a 10-year period (2008 to 2017), we collected 138 cases with clinical suspicion of sporotrichosis. No control (healthy) group was included. All of the patients received 0.1 mL of intradermal sporotrichin M (mycelia), which was injected into their forearm [12]. The reading was ascertained at 48 h, using the same criterion as for the tuberculin skin test. The skin test was considered to be positive when induration was present and was equal or higher than 0.8 cm [11].

The sporotrichin antigen used in this study was a filtrate culture medium concentrate of the fungal mycelial phase, which was prepared by modifying the method of Arenas and Toriello [12]. Briefly, a semisynthetic culture medium was incubated for two weeks at 28 °C; cultures were thimerosal treated, filtrated, centrifuged, dialyzed, and concentrated to a final protein concentration of 10 µg protein/0.1 mL [11]. The antigen was prepared from reference strain *S. schenckii* EH–143 from the Laboratory of Basic Mycology, Department of Microbiology and Parasitology, Faculty of Medicine of the Universidad Nacional Autónoma de México. This fungal strain has been deposited in the fungal collection of the laboratory registered in the World Federation for Culture Collections with the code BMFM–UNAM 834.

Biological samples of the suspected cases of Sporotrichosis were cultured on Sabouraud dextrose agar and Sabouraud with antibiotics (0.01% cycloheximide and 0.001% chloramphenicol). Fungal cultures obtained from patients were identified by macromorphology (culture colonies that are initially creamy colored become wrinkled, membranous, and develop a brown-black color) and micromorphology (thin, septate hyphae, with sporulating conidiophores that suggest a flower head), which were further confirmed through molecular biology by DNA extraction of each strain and further gene sequence of a partial calmodulin gene [13].

### 2.2. Data Analysis

Sample size was calculated assuming a sensitivity of 80% and a specificity of 100% for the sporotrichin test. The standard normal deviation for two-sided α with a 95% confidence interval was 1.96. Sensitivity values of the sporotrichin test were determined using culture as a gold standard. All of the statistical analysis was performed using SPSS version 23 for Windows (Chicago, IL, USA).

## 3. Results

Among the 138 cases with clinical suspicion of sporotrichosis included in this study, 56 recorded positive responses to sporotrichin, and among those 56 cases, 4 (7.1%) were false-positives. These four patients came from high endemic areas, and one of them had already contracted the disease.

Furthermore, 55 cases of sporotrichosis were tested through cultures, out of which 52 were recorded to be positive for sporotrichin (94.5%) and the remaining three (5.5%) were false-negatives, which corresponded to disseminated cutaneous sporotrichosis (two cases with chronic alcoholism and one with C3-stage HIV-AIDS). The distribution in terms of gender was 34 male (62%) and 18 female (38%) cases, whereas the distribution in terms of their ages consisted of 17 children and adolescents under the age of 18 (31%), and 38 adults (69%). The isolates were identified as *Sporothrix schenckii* 54/55 (98%) and 1/55 (2%), which corresponded to *Sporothrix globosa*.

**Figure 1:**
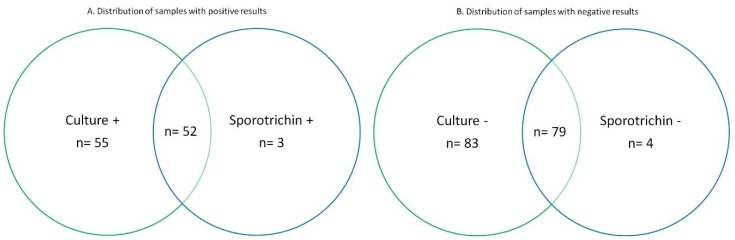
Venn diagram showing the distribution of positive laboratory results. + means positive, and − means negative.

We calculated a sensitivity of 94.5% and a specificity of 95.2%, where false-positives were observed in 7.1% of the cases. Out of the tested, 55 could be confirmed by culture, and could therefore be considered true positives (Sporotrichin positive *n* = 52) (Figure 1).

**Figure 2:**
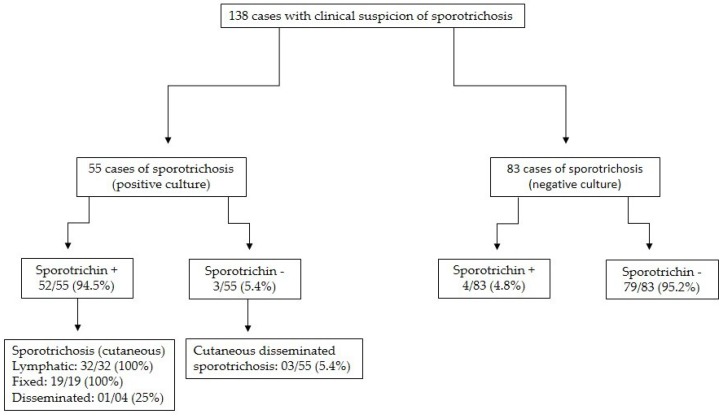
Flowchart of the cases studied. + means positive, and − means negative.

**Figure 3:**
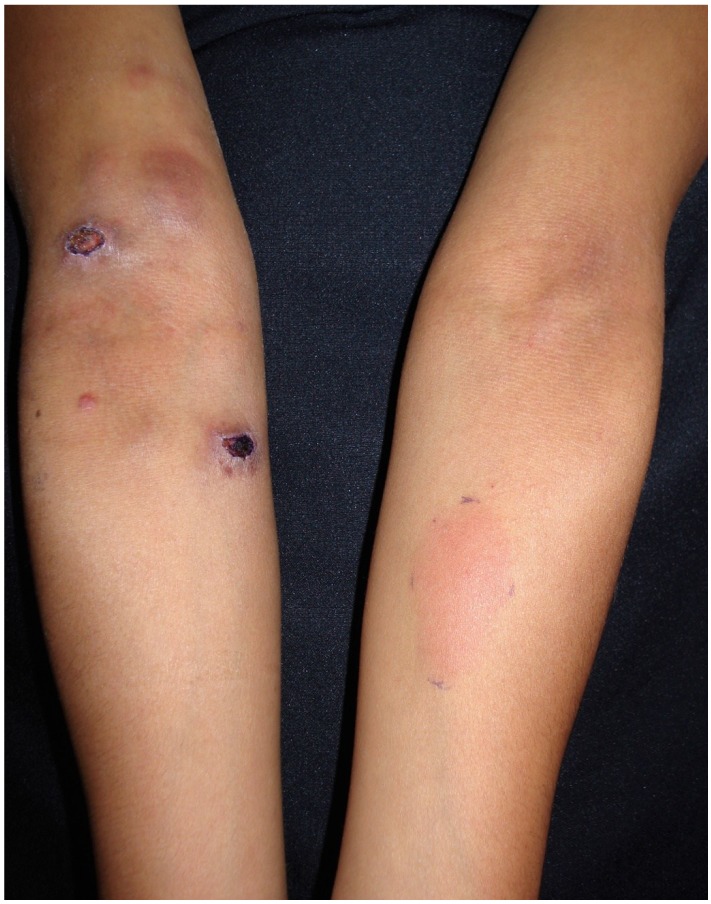
Cutaneous lymphangitic sporotrichosis and its hyperergic response (>2 cm induration and erythema) to sporotrichin M.

The flowchart of the cases has been presented in Figure 2. In Table 1, the types of clinical forms and their positive response to sporotrichin have been reported. (Figure 3). Moreover, Table 1 depicts the clinical types according to the intensity of their response to sporotrichin.

## 4. Discussion

Sporotrichin was obtained from *S. schenckii* (ss) strain for the first time in 1947 by González-Ochoa [9,10]. This antigen was developed from a crude extract of the fungus’ mycelial phase, and it was primarily used as an intradermal reaction, i.e., an aspect that measures cellular immunity [2,3,12,14]. Currently, the production of the antigen is made from minimal culture media, i.e., simple metabolites and salts, so that the fungus elaborates less contaminated antigenic structures; especially if they have to be compared to the first ones that integrated the culture media with large peptides. Hence, the fungus tends toward coarser antigens. As *S. schenckii* is a thermodimorphic fungus, it is also possible to extract the antigen during the yeast-like phase [1,3,12,14].

The antigens produced by *S. schenckii* are different from the rest of the primary and opportunistic pathogenic fungi antigens due to a rhamnose-containing fraction that provides them with a greater specificity. Other studies have shown that S. schenckii’s antigens are composed of peptide-rhamnomannans [1,3,12,15,16,17].

The use of the sporotrichin skin test is not accepted in most European countries or in the United States, because they consider it a lack of standardization, although fragments of the fungus are used in certain cases [2,3]. In our experience, similar to other countries, the sporotrichin skin test provides a fundamental tool to know the patient’s cellular immune response and for epidemiological studies of Sporotrichosis [2,3,11,14].

Conducting an analysis on the results of our study, it is observed that the effectiveness of sporotrichin to diagnose sporotrichosis was 93%. This data is comparable with a few reported series. For instance, Kushamara [18] reported it to be 89.3%, Itoh [19] reported it to be 96.3% in Japan, and Rocha-Posada [20] reported it as 91.4% in Colombia. To elaborate, most series provide similar data, which is higher than 90% on most occasions. Considering the analysis of false-positive cases with similar dermatoses and negative cultures, they only occurred in four cases (7.1%). It is important to mention that all of these cases came from high endemic regions (northern Sierra de Puebla) [11]. Moreover, in 75% of the cases (three out of four patients), they were considered to be positive reactors, who were probably in constant contact with the fungus, and in one case, the patient had been previously affected with the disease. Thus, it was considered that the positive response maintains long, specific hypersensitivity, and possibly acts as a protector.

The specificity of the response was 3/55 (94.6%), which represents the false-negative, verified cases of sporotrichosis (by culture), and the three cases corresponded to the disseminated skin cases, with the associated immunosuppression factor. Out of these three, two were due to chronic alcoholism, and one was more in-phase C3 AIDS-related, with the count of CD4 lymphocytes being below 200 cells. Moreover, these cases are a part of a recent report concerning this type of sporotrichosis [21,22].

According to the data pertaining to the cases of sporotrichosis included in this study, they are similar to several reports that show cases with a predominance of males (62%) and adults (69%), in addition to having a predominance of *S. schenckii* (98%). This has been the most commonly reported species in Mexico and other regions of Central and North America. Furthermore, a predominance of cutaneous-lymphatic sporotrichosis has been observed [1,2,3,4].

Table 1 shows that all of the cases included in the lymphangitic and fixed forms were positive, which represent the forms that always respond to the antigen. Meanwhile, in the disseminated cases, it was only positive in 25% of the cases (1 of 4), and corresponded with the reports of a chronic patient affected with diabetes [21].

We consider that the clinical form of sporotrichosis is dictated by the immunity of the patient, and not the virulence of the fungus. This observation is reinforced by the data that we have presented in Table 1, according to which the majority of the normal-positive responses were observed in the cutaneous-lymphangitic form (68.7%), while the majority of the hyperergic-positive responses occurred in the cutaneous-fixed form (89.5%). Therefore, it can be concluded that the latter occurs in patients with better immune responses. In contrast, almost all of the disseminated cases (75%) were negative, which could suggest a patient’s inefficient cellular immune response, taking into consideration that skin tests express this retarded hypersensitive reaction [3,21,22].

Our general commentary: with regard to false positives, these correspond to patients who live in endemic areas and who are probably in constant contact with the antigen, or cases that had the disease and preserve immunological memory, and there is no corresponding failure of the intradermal antigen. In the same way, false negative cases correspond to patients with different degrees of immunodepression and neither does the antigen fail, nor the patient’s immune system. This has been previously reported [2,3,11,21].

Additionally, the duration of the sporotrichin reactivity is unknown. However, for some cases that we have been able to study for years, we have had reports stating that the response is still positive after more than 20 years. It is interesting to note that a case that we observed several years ago developed lymphangitic sporotrichosis and presented a fixed form years later, probably owing to a better immune response [3].

We consider that the intradermal reaction to sporotrichin is still a great aid in the diagnosis of the disease. It provides us with highly suggestive data (>90%) that can be attained within 48 h, and it is suggested that it is always correlated with fungus isolation. The positivity data also allows us to form an idea of the patient’s immune status, and this point is also of interest in case a treatment with potassium iodide is implemented, as this is considered to be an immune stimulant and would function better in positive-sporotrichin cases.

The intradermal reaction to sporotrichin has also been used to determine the positive reactors, as well as the first contact with the fungus, without developing the disease. This allows us to know areas of high endemicity. For such cases, the yeast form of the antigen, which is obtained more quickly, is much more useful. Examples of these instances include a few reports provided in Mexico [17,23], the United States [24], Brazil [25], and India [26].

## 5. Conclusions

The intradermal reaction to mycelial phase sporotrichin is an excellent antigen for skin testing and epidemiological studies. In this work, we found a sensitivity of 94.5% and a specificity of 95.2%, where the false-positives were limited to 7.1%. It continues to be a rapid test that is auxiliary in terms of the diagnosis, and allows us to ascertain the patient’s cellular immune status.

## Figures and Tables

**Table 1:** Clinical types of sporotrichosis and response to the sporotrichin M skin test.

	Clinical Form		
	Cutaneous Lymphatic	Cutaneous Fixed	Cutaneous-Disseminated
No. of patients	32 (58.2%)	19 (34.6%)	4 (7.2%)
Positive response to sporotricin M	32/32 (100%)	19/19 (100%)	1/4 (25%)
Normal-positive reaction (0.5–2 cm)	22/32 (68.7%)	2/19 (10.5%)	1/4 (25.0%)
Hyperergic-positive reaction (>2 cm)	10/32 (31.3%)	17/19 (89.5%)	None
